# Biocompatibility and efficacy of prostatic urethral lift in benign prostate hyperplasia: an in vivo and in vitro study

**DOI:** 10.1038/s41598-023-40889-w

**Published:** 2023-08-24

**Authors:** Yuqi Xia, Tianhui Yuan, Wei Zou, Haoyong Li, Jinzhuo Ning, Yuan Ruan, Lizhe Xu, Weimin Yu, Fan Cheng

**Affiliations:** Department of Urology, Renmin Hospital of Wuhan University, Wuhan, China

**Keywords:** Prostate, Experimental models of disease, Preclinical research, Translational research, Urinary tract obstruction, Urogenital reproductive disorders

## Abstract

The study aimed to assess the biocompatibility and efficacy of a prostatic urethral lift (PUL) for benign prostatic hyperplasia (BPH). Human BPH-1 cells were co-cultured with implant anchors and sutures, and cytotoxicity was measured. Scanning electron microscopy (SEM) was used to observe adhesion and growth of cells and to evaluate implant biocompatibility. Fifteen male beagle dogs were randomly assigned to the surgical (n = 9) or sham-operated (n = 6) groups. The surgical group underwent cystotomy, and PUL was used to insert two implants in each lobe of the prostate to compress the enlarged prostate and dilate the urethra; the sham group underwent cystotomy without implant insertion. Compared with the control group, no significant difference in cell viability among the groups with different co-culture times of implant anchors and sutures (P > 0.05) was observed. SEM revealed good adhesion and growth of prostate cells on the implants. Improvements in urine flow rates remained stable at 7, 28, and 180 days after surgery, and the urethral diameter in the prostate region was significantly increased compared with that before surgery. PUL is a biocompatible and effective treatment for BPH, improving the urine flow rate without causing inflammation, tissue damage, or cytotoxic effects. Here, the basis for further PUL application was provided.

## Introduction

Benign prostatic hyperplasia (BPH) is a common condition among older men, with a lifetime prevalence of 26.2%^1^, ranging from 14 to 30% in men aged 50 years or older^2,3^. BPH is characterised by the enlargement of the prostate gland, which can lead to a range of urinary symptoms, including weak urinary stream, frequency, urgency, and incomplete emptying^2^. In severe cases, BPH can lead to acute urinary retention, urinary tract infection, and renal impairment. These symptoms can considerably affect the quality of life by causing distress, embarrassment, and loss of confidence. Thus, BPH is associated with a significant economic burden due to the costs associated with managing the condition.

Primary treatment methods for BPH include medical therapy, minimally invasive procedures, and surgical interventions^4^. Medical therapy involves the use of alpha-receptor blockers, 5-alpha-reductase inhibitors, or a combination thereof. Transurethral resection of the prostate (TURP) is the first choice for patients with severe symptoms or in whom medical treatment has failed. However, TURP cannot meet the needs of all patients, especially those who need to preserve normal ejaculation function^5^. Currently, emerging treatments such as transurethral microwave thermotherapy, laser therapy, and water Jet-Hydrodissection are being increasingly used in the treatment of BPH^6^. Continuous improvements in treatment methods and the development of new surgical procedures are necessary.

A prostatic urethral lift (PUL) is performed under local or general anaesthesia to create a continuous anterior prostatic urethral channel by inserting small, permanently sutured implants under cystoscopic guidance using a special disposable device that compresses the lateral lobes^7^. The implant consists of three parts: a capsular tab, a urethral end piece, and sutures. The capsular tab is fixed outside the prostate envelope, and the urethral end piece is fixed in the urethra of the prostate segment; sutures are made using an adjustable polyester monofilament. Over the past decade, several large multicentre clinical studies have validated PUL as a safe and effective therapy for patients with BPH who want to maintain sexual function and rapidly improve their symptoms^5,8–10^. PUL is recommended by the European Association of Urology and American Urologic Association guidelines as a minimally invasive treatment option^11^. Therefore, it is essential to investigate its biocompatibility for use as a permanent implant. No in vitro or long-term animal studies have been conducted to verify this biocompatibility.

In the present study, we performed in vitro biocompatibility assays and long-term animal studies to evaluate the biocompatibility and efficacy of PUL implants, which are non-toxic materials with good compatibility and stable efficacy.

## Materials and methods

### Cytotoxicity assessment via CCK-8 assay

The CCK-8 kit was obtained from the Tong Ren Chemical Research Institute, Japan. The human prostate hyperplasia cell line (BPH-1) and RPMI-1640 medium were obtained from Wuhan Service Biotechnology Co. The capsular tab, urethral end piece, and sutures were sterilised and added to RPMI-1640 complete medium at a ratio of 0.2 g/ml and incubated at 37 °C for 48 h to prepare the implant extracts.

A suspension of 2 × 10^3^ BPH-1 cells, in a volume of 100 μL, was seeded into 96-well plates and incubated for 12 h in a 5% CO_2_ environment to allow for adhesion. The medium was then replaced with the implant extract. The control group was maintained in RPMI-1640 complete medium, whereas the experimental group was exposed to the capsular tab, urethral end piece, and suture extracts, each with three replicates per group. According to previous studies^12,13^, following incubation for 1, 3, 5, and 7 days, 10 μL of CCK-8 reaction solution was added to each well. The samples were then incubated at 37 °C for 1 h and Absorbance, A (Absorbance, A) was measured at 450 nm using a multifunctional enzyme marker (Perkin Elmer, USA). The relative cell viability was determined as [(A sample group)/(A control group) × 100].

### Scanning electron microscopy (SEM)

A suspension of 1 ml containing 1 × 10^5^ BPH-1 cells was inoculated into a sterilised 24-well plate fitted with a sterilised capsular tab and urethral end piece. The cells were incubated in a 5% CO_2_ atmosphere for 5 days. Upon removal of the growth medium, the cells were gently washed three times with phosphate-buffered saline (PBS). Subsequently, the cells were fixed with 2.5% glutaraldehyde and were sequentially dehydrated in anhydrous ethanol at concentrations of 30, 50, 80, 90, 95, and 100%. The cells were lyophilised overnight, coated with gold, and imaged using a scanning electron microscope (ZEISS, Germany).

### Phalloidin staining

A Phalloidin staining kit was obtained from Shanghai Yisheng Biotechnology Co. A cell suspension (2 ml containing 4 × 10^5^ BPH-1 cells) was inoculated into a sterilised 6-well plate fitted with sterilised coverslips. The cells were incubated in a 5% CO_2_ atmosphere for 12 h to allow adherence to the coverslips. The growth medium was then replaced with implant extracts. The control group was incubated in the RPMI-1640 complete medium, whereas the experimental group was exposed to the capsular tab, urethral end piece, and suture extracts. After an additional 5-day incubation period, the medium was removed, and the cells were gently washed three times with PBS. The cells were fixed with 4% paraformaldehyde for 20 min. After washing with PBS, the cells were stained with ghost pen cyclic peptide working solution and DAPI staining solution for 30 and 10 min, respectively. The cells were imaged using a fluorescence microscope (Olympus, Japan), and five random fields of view were selected for analysis. The positive expression area of the cytoskeleton was quantified using ImageJ software (1.46r, NIH, USA) and was calculated as the ratio of the positive staining area for the ghost cyclopeptide to the total area of the field of view, expressed as a percentage.

### Animals

The present study adhered to the guidelines established by the Chinese Animal Protection Committee and was approved by the Laboratory Animal Welfare and Ethics Committee of the Renmin Hospital of Wuhan University (approval number: WDRM-yzc20201018). The experiment was conducted following ARRIVE guidelines (https://arriveguidelines.org). Beagle dogs with spontaneous BPH were selected^14^. Fifteen male beagles with a mean age of 7 years and a weight of 17–26 kg, and certified health status, were selected for this study.The average prostate volume was 17.95cm^3^,which meets the criteria for BPH. Before experimentation, the animals underwent a 7-day acclimatization period. Subsequently, the animals were housed in individual cages measuring > 0.74 m^2^ and maintained at an ambient temperature of 23 ± 2 °C, relative humidity of 50 ± 10%, ventilation frequency of 10–15 times/hour, and a 12-h light cycle (commencing at 8 am and terminating at 8 pm) with an illumination level of 150–300 lx. The dogs were provided autoclaved food and water. The animals were randomly divided into two groups, with nine animals undergoing surgery, and six serving as sham-operated controls.

### PUL for animals

**Figure 1:**
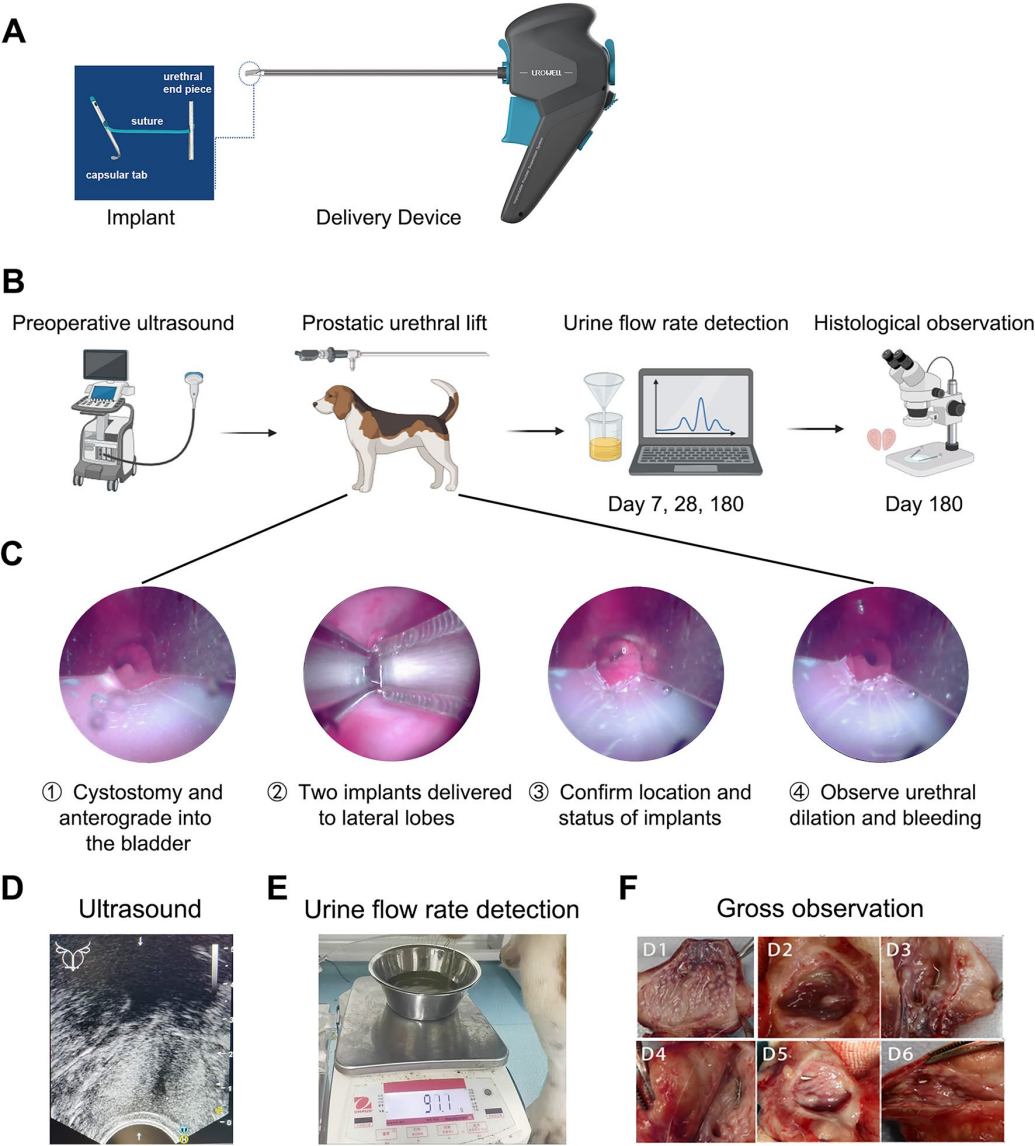
Experimental procedures and surgical steps for PUL in animal models. (**A**) PUL surgical system. (**B**) Flowchart depicting the experimental procedures in the animal models. (**C**) Surgical steps of PUL procedure under cystoscopic view. (**D**) Preoperative ultrasound detection. (**E**) Urine flow rate detected by uroflowmeter. (**F**) Gross observation of prostate.

The PUL surgical system (PL100, *Urowell*, China) used in the current study has the same mechanism as the Urolift® system commonly used^15^, but the internal structure of the instrument is different. During the surgical procedure, the animals were placed in the supine position and anaesthetised by intravenous administration of 35 mg/kg pentobarbital. Because of the presence of the penile bone, a retrograde cystoscope was difficult to perform^16^. Thus, the operation was performed using abdominal and anterograde approaches to the bladder. The skin on the abdomen was prepared, sterile towels were laid, the penis was exposed on one side, and an incision was made on the lateral aspect of the rectus abdominis muscle. Cystostomy was performed through the abdomen, and the cystoscope entered the bladder in a cascade. PUL surgical system was utilized to insert micro-implants into the left and right lobes of the prostate (2–3 o'clock and 9–10 o'clock direction of the most obvious lobe enlargement, at a distance of roughly 0.5–1 cm from the bladder neck opening) to compress and suspend the enlarged gland, and to dilate the urethra. Subsequently, implant fixation was confirmed, and urethral dilatation and bleeding were observed (Fig. 1A–D). Subsequently, the cystostomy was removed. In the sham-operated group, only cystostomy was performed and no implants were inserted. Postoperative antibiotic prophylaxis was administered. At the end of the experiment, the beagles were euthanised with excess pentobarbital sodium (intravenous injection, 125 mg/kg) and the prostates were removed.

### Animal ultrasound

Ultrasound imaging and volume measurements were performed by using a transrectal ultrasound imaging system. A low-frequency probe was placed on the lower abdominal skin near the root of the penis, while a 3.5 MHz convex array probe was used to display prostate images. The measurements were performed using the ellipsoidal approximation method (Fig. 1D).

### Urine flow rate assay

The animal was placed on a homemade cuboid frame and the bladder was filled with 250 ml of saline at a rate of 10 ml/min. A uroflowmeter was placed under the penis of the dog and the urine flow rate was calculated by plotting the flow rate and time curve (Fig. 1E).

### Gross observation and histopathology staining

The diameter of the prostatic urethra was measured using a cystoscope by referring to the width of the surgical instruments before PUL and 28 and 180 days after PUL. The beagles were euthanised, and the prostates were removed on days 28 (n = 3) and 180 (n = 6) after PUL. The implant structure and fixation were also observed (Fig. 1F). Prostate tissues were then embedded in paraffin and were cut into 5 μm slices, after fixation in 4% paraformaldehyde. Following general dewaxing and hydration, the slices were stained with haematoxylin and eosin (HE). Tissue fibrosis was evaluated using Masson’s trichrome staining. The protein expression levels of TNF-α, and Fibronectin were analysed using immunohistochemical staining. Antibodies (i.e., TNF-α [60291-1-Ig] and fibronectin [15613-1-AP]) were purchased from Proteintech (USA). Tissue sections were stained for pathological analysis after baking and dewaxing.

### Statistical analysis

Data are presented as mean ± standard deviation, and all statistical analyses were conducted using SPSS (version 17.0; SPSS Inc., Chicago, IL). Paired Student's t-tests were performed to compare the pre- and post-treatment values, while differences between the two groups were analysed using analysis of variance with Greenhouse–Geisser correction. Statistical significance was set at P < 0.05.

## Results

### In vitro cytotoxicity assay of implants in BPH-1 cells

**Figure 2:**
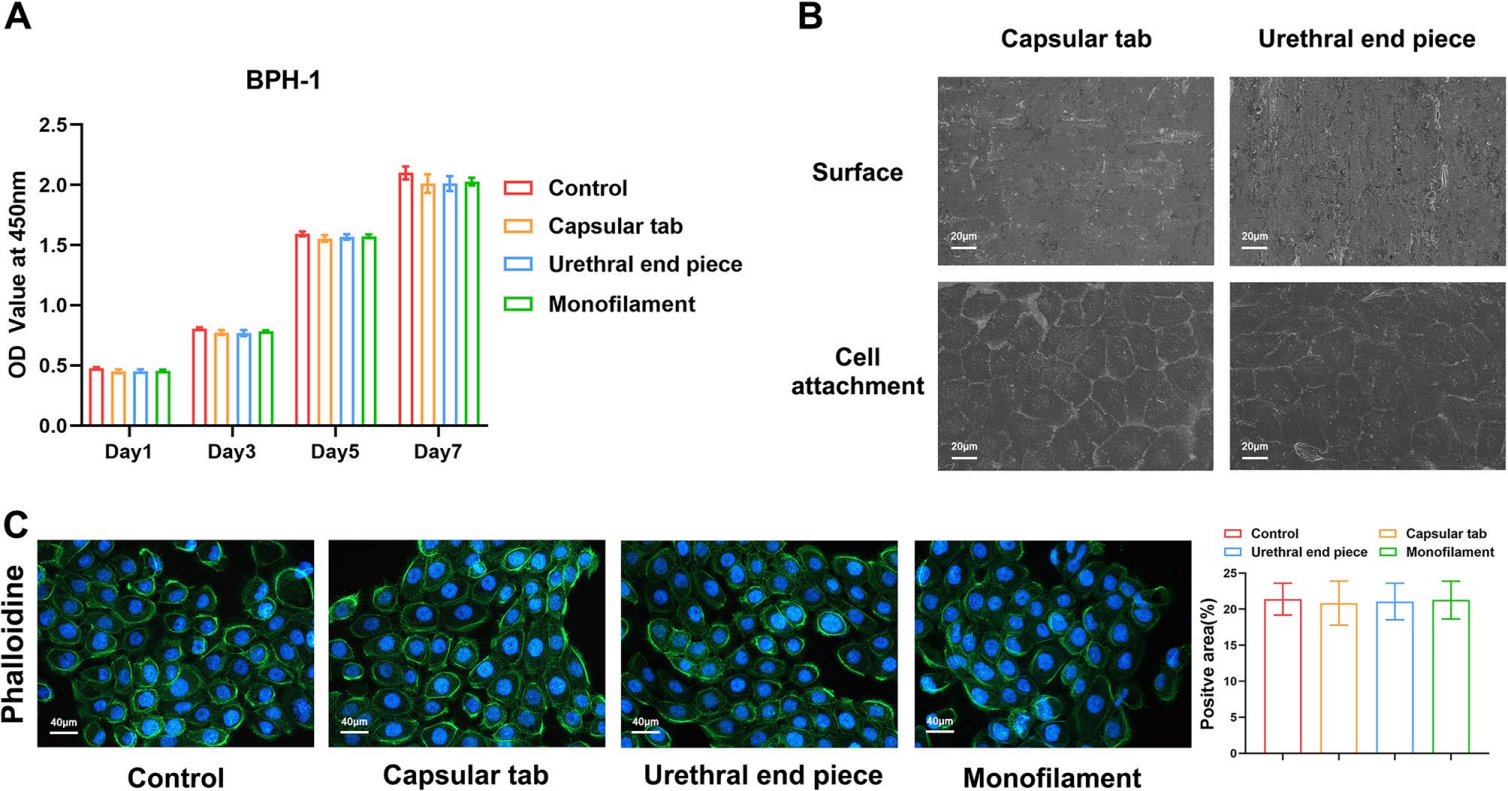
Biocompatibility assays of PUL implants in BPH-1. (**A**) Cytotoxicity assessed by the CCK-8 assay. (**B**) Surface topography and cell attachment of the capsular tab and urethral end piece observed using SEM (500 ×). (**C**) Cytoskeleton stained with phalloidin and visualized under fluorescence microscopy (400 ×).

The CCK-8 results showed no significant difference in the A value between the experimental and control groups when BPH-1 cells were co-cultured with the capsular tab, urethral end piece, and monofilament extracts on days 1, 3, 5, and 7 (F = 2.865, P = 0.104; F = 2.887, P = 0.102; F = 1.692, P = 0.245; F = 1.584, P = 0.268, respectively). The relative survival rates of the capsular tab, urethral end piece, and suture of the prostate implant on days 1, 3, 5, and 7 were 94.49%, 94.76%, and 95.24%; 95.91%, 95.16%, and 96.99%; 97.55%, 98.37%, and 98.74%; and 95.78%, 95.75%, and 95.75%, respectively (Fig. 2A).

### SEM observation of cell adhesion on implants

SEM was used to observe the adhesion of BPH-1 cells on the surface of the capsular tab and urethral endpiece and the cells on the implant after co-culture for 5 days. BPH-1 cells grew well on the first and second anchors and had a normal square shape, indicating that the implant did not affect cell adhesion and had good biocompatibility (Fig. 2B).

### Phalloidin staining of BPH-1 cells with the implant extracts

Phalloidin staining was used to observe the distribution of microfilaments in BPH-1 cells co-cultured with the capsular tab, urethral endpiece, and monofilament extracts of the prostate implant for 5 days. The positive expression areas (%) of the cell skeleton were not significantly different from those of the control group (F = 0.041, P = 0.988) (Fig. 2C), indicating that the implant did not damage the cell skeleton and had good biocompatibility. The results demonstrated that the prostate implant, including the capsular tab, urethral endpiece, and monofilament, had good biocompatibility and did not exhibit any cytotoxic effects on BPH-1 cells.

### Application of PUL in beagle dogs with BPH

Preoperative measurement of prostate volume was performed using ultrasound (the prostate volume of the surgical group was 18.58 ± 4.88 cm^3^, while that of the sham group was 17.08 ± 5.32 cm^3^), followed by PUL surgery, and postoperative measurement of urine flow rate at 7, 28, and 180 days. After 180 days, the animals were euthanised and the prostates were removed for gross observation and histological staining (Fig. 1A). Using an implant delivery device under cystoscopy, we inserted two implants into the lateral lobes of the prostate, confirmed the position and status of the implants, and observed urethral dilation and bleeding (Fig. 1B). After PUL treatment, all dogs recovered from the lower urinary tract symptoms caused by BPH, including dysuria and frequency. The dogs in the sham group remained clinically stable. Two dogs in the surgical group experienced minor bleeding during surgery and recovered immediately following the procedure. No complications were reported in the other dogs in the surgical group. The diameter of the prostatic urethra was significantly widened by an average of 15.3 ± 2.9 mm and 13.4 ± 1.1 mm at 28 and 180 days postoperatively, respectively.

**Figure 3:**
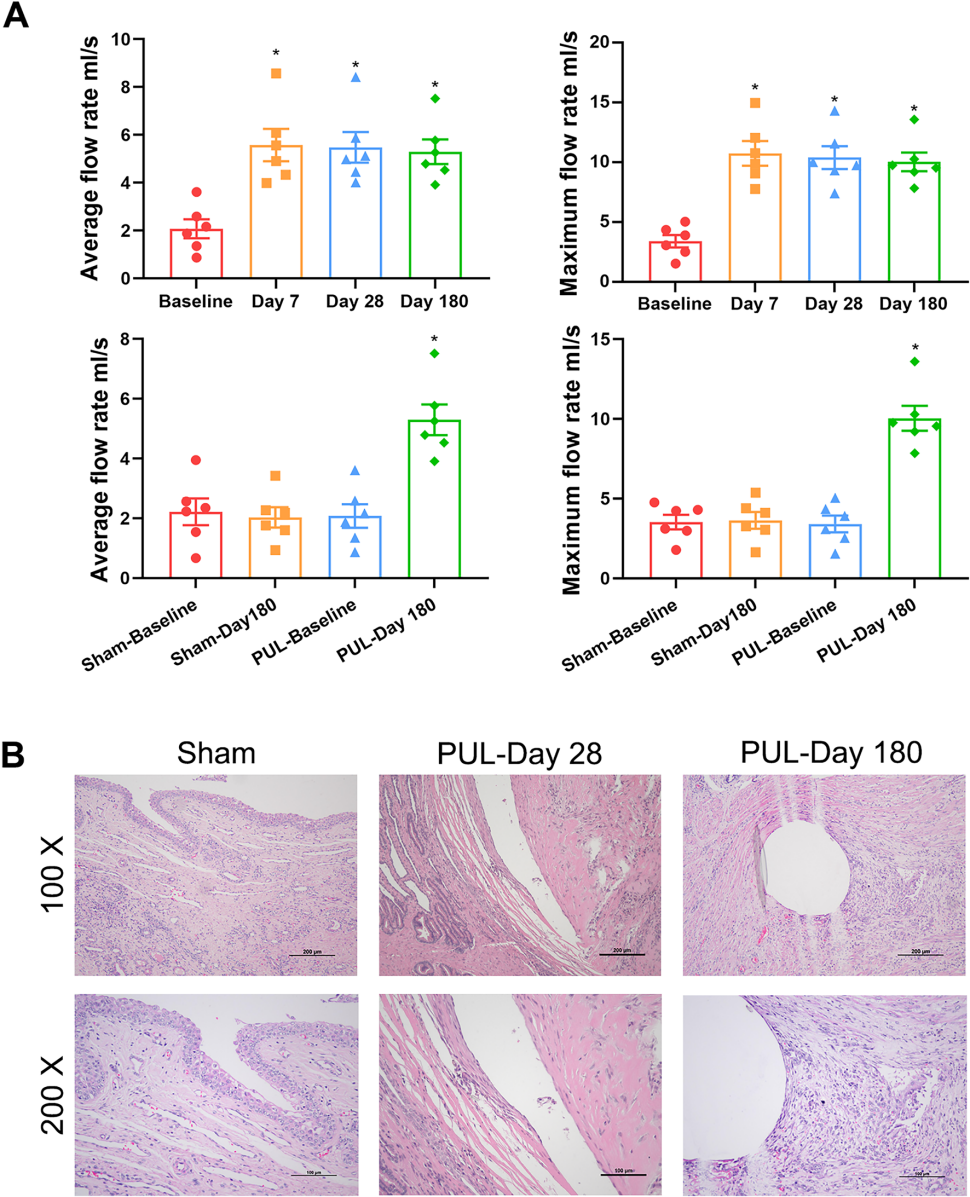
Urine flow rate and HE staining of prostate tissue in Beagles after PUL. (**A**) Mean and maximum urine flow rates for the sham surgery group, baseline, and postoperative days 7, 28, and 180 for PUL treatment. (**B**) HE staining of prostate tissue for the sham surgery group, and PUL treatment at 28 and 180 days postoperatively. *P < 0.05 vs. Baseline.

The preoperative urine flow rate was used as the baseline. At 7 days postoperatively, the average (5.57 ± 1.66 ml/s) and the maximum (10.76 ± 2.52 ml/s) flow rates of the surgical group improved significantly from the baseline measurements (2.08 ± 0.96 ml/s and 3.41 ± 1.28 ml/s) (P < 0.05), and this improvement remained stable at 28 and 180 days postoperatively (P > 0.05) (Fig. 3A). In addition, no significant changes in the average and maximum urine flow rates in the sham group were observed 180 days postoperatively (P > 0.05) (Fig. 3A). Interestingly, beagles in this study showed enlargement of both the median and lateral lobes. In all dogs in the surgical group, the obstruction was relieved and PUL was effective.

**Figure 4:**
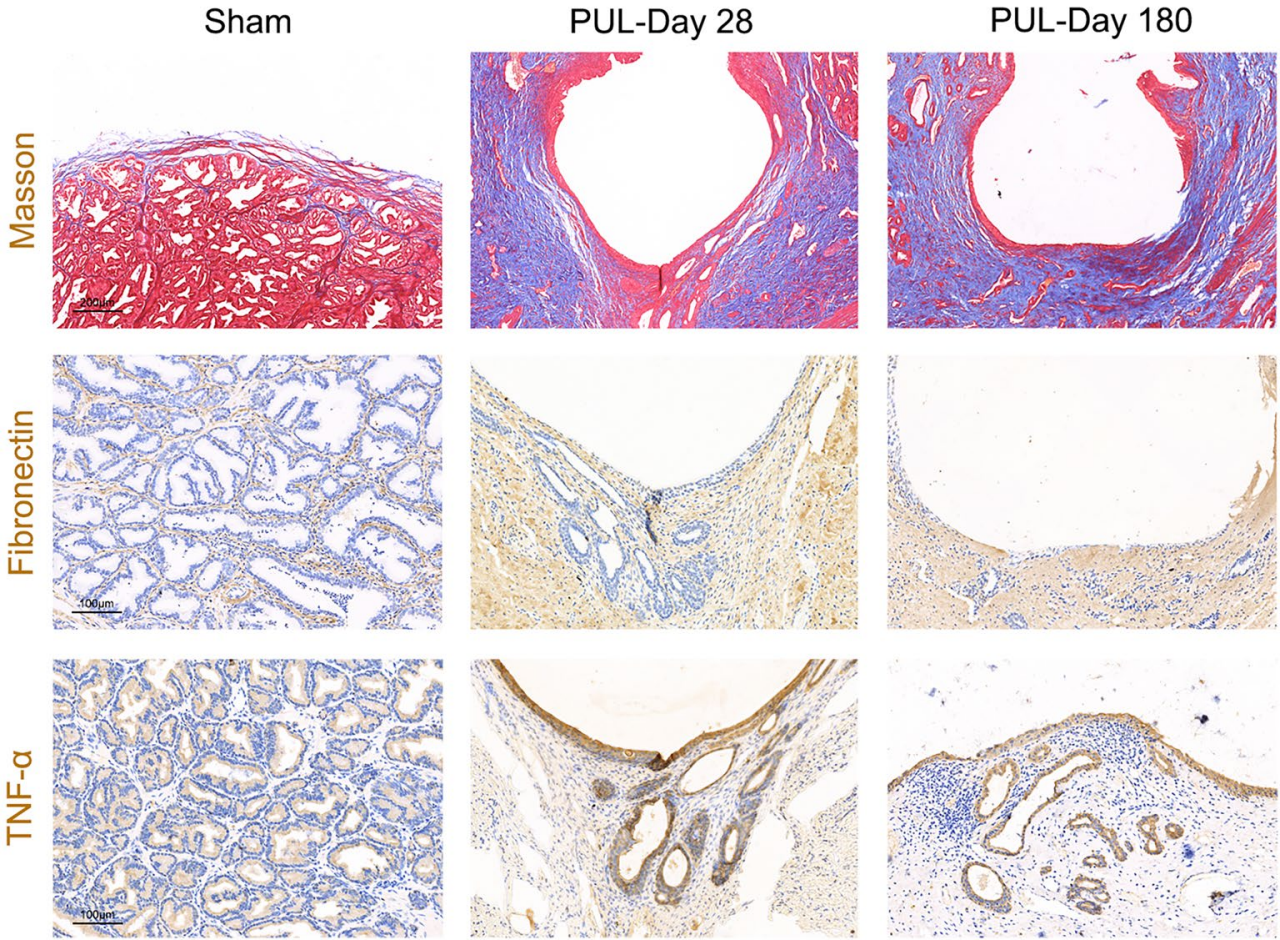
Masson and immunohistochemical staining of prostate tissue in Beagles after PUL. (**a**) Masson staining assessed the fibrosis of prostate tissue for the sham surgery group, and PUL treatment at 28 and 180 days postoperatively (100 ×). Immunohistochemical staining evaluated the TNF-α and Fibronectin expression of prostate tissue for the sham surgery group, and PUL treatment at 28 and 180 days (200 ×).

Gross observations revealed that the implants were structurally intact and fixed to the urethra of the prostatic segment, which was dilated and unobstructed (Fig. 1E). HE, Masson and immunohistochemical stainings of the prostate tissue showed slight inflammatory cell infiltration, collagen fibre deposition, TNF-α and fibronectin expression 28 days after PUL. A small amount of connective tissue hyperplasia and stable scar formation were observed around the implant at 180 days after PUL. No glandular hyperplasia, infection, haemorrhage, or new growth was observed at 28 or 180 days postoperatively. The results indicated the good biocompatibility and durability of the implants (Figs. 3B, 4).

## Discussion

Although Urolift ® is widely used, related studies have mainly focused on clinical outcomes. The biocompatibility and efficacy of PUL in vivo and in vitro, particularly in histopathological studies, have rarely been reported. This study verified the biocompatibility and efficacy of the PUL implants using in vitro biocompatibility assays in BPH-1 cells and long-term animal experiments. BPH-1 was co-cultured with the capsular tab, urethral end piece, and monofilament extracts, and its biocompatibility was assessed through cytotoxicity assays, cell adhesion experiments, and cell skeleton staining.

In vivo experiments were conducted by performing PUL surgery in beagle dogs. Urine flow rate measurements, gross observations, HE, Masson’s trichrome, and immunohistochemical stainings were performed to compare the effectiveness and safety of PUL. Our results indicated that the implant was non-toxic to prostate cells, and human prostate hyperplasia cells adhered and grew normally on the capsular tab and urethral end piece of the implant, which did not affect cell skeleton distribution. Following PUL, a marked increase in urinary flow rate was observed in beagle dogs. Additionally, the structure of the implant was intact: no loosening or falling off was observed, and the urethra was continuous and unobstructed without an accompanying inflammatory response to the implant 180 days postoperatively, indicating good efficacy, biocompatibility, and durability.

The capsular tab was made of nitinol, the urethral end piece was made of medical stainless steel, and the sutures were made of an adjustable polyester monofilament. These materials are widely used in biomedicine and demonstrate good biocompatibility in tissues such as the bronchi and arteries^17^. Hamann et al.^18^ have reported that human bone marrow stromal cells adhered and proliferated on the surface of a nickel-titanium alloy, and immunofluorescence staining showed the expression of cell skeleton proteins such as F-actin and Vinculin, indicating that nickel-titanium alloy materials support cell adhesion and cell skeleton formation. Shang et al.^19^ placed human coronary artery endothelial cells on the surface of a nickel-titanium alloy and observed that the cells adhered, grew, and migrated normally through laser confocal microscopy and expressed endothelial cell markers such as Vinculin and Platelet endothelial cell adhesion molecule-1, suggesting that nickel-titanium alloy materials can maintain a typical endothelial cell phenotype. Medical stainless steel has good mechanical and corrosion resistance, is inexpensive and widely used in surgical implants for cardiovascular, orthopaedic, and maxillofacial surgeries^20^. Moreover, a clinical study of 206 patients has shown no recurrent urinary tract infections or inflammatory reactions in the prostate 5 years after PUL^8^. Consistent with the above findings, the present study found that PUL implants are nontoxic and compatible with in vivo and in vitro materials.

Several multicentre clinical studies have shown that the International Prostate Symptom Score(IPSS) and maximum urine flow rate improved significantly after PUL^8,21–23^ and that IPSS and maximum urine flow rate at 3 and 5 years after PUL were not significantly different from those at 1 year after surgery^8^. Consistent with the long-term results of our study, PUL is an effective and minimally invasive procedure for the treatment of BPH.

Woo et al.^24^ observed four patients (21%) that underwent TURP retreatment within 1 year after PUL, and they have reported no difficulty in cutting the suture of the PUL device during the operation. Histopathological examination of the suture site confirmed good histocompatibility of the implanted device, and no reports of implant scaling were found. In the LIFT study^25^, cystoscopy was performed 1 year after surgery, and no scales were found in all implants located in the prostate. Consistent with the above results, the present study found that the structure of the implant was intact and no loosening or falling off was observed 180 days postoperatively. PUL improved the urine flow rate without causing inflammation in long-term experiments in beagle dogs, confirming the stability, efficacy, and durability of PUL.

PUL surgery can be performed under local anaesthesia, is simple and effective, and has few postoperative complications while preserving the sexual function of patients, making it a promising minimally invasive procedure. The results of this study provide a basis for further promotion and application of PUL surgery. A limitation of this study was that residual urine volume and other objective parameters related to BPH were not investigated. Moreover, because the prostate gland continues to grow with age, postoperative enlargement of the gland and decreased material quality may require a longer follow-up. Coating implant surfaces to further optimise biocompatibility and prevent postoperative infections, stone formation, and material corrosion is a promising research direction.

## Conclusion

Our findings demonstrated that PUL is a biocompatible and effective treatment for BPH. PUL implants are noncytotoxic and have good biocompatibility with the prostate. Long-term experiments in beagle dogs showed that PUL can dilate the urethra and improve the urine flow rate without causing inflammation or tissue damage, which provides the basis for further application of PUL.

## Data Availability

The datasets used and analyzed during the current study available from the corresponding author on reasonable request.
